# A Study Using Visual Art Methods to Explore the Perceptions and Barriers of Self-Management in Children and Adolescents with Lymphedema

**DOI:** 10.1089/lrb.2018.0075

**Published:** 2019-04-22

**Authors:** Christine Moffatt, Aimee Aubeeluck, Elodie Stasi, Sandrine Mestre, Sara Rowan, Susie Murray, Isabelle Quéré

**Affiliations:** ^1^ School of Social Sciences, Nottingham Trent University, Nottingham, United Kingdom.; ^2^ Department of Vascular Medicine, EA2992, CHU Saint Eloi, University of Montpellier, Montpellier, France.; ^3^ School of Health Science and Medicine, University of Nottingham, Nottingham, United Kingdom.; ^4^ Center of Research of Immunopathology and Rare Diseases—Coordinating Center of the Network for Rare Diseases of Piedmont and Aosta Valley, Turin, Italy.; ^5^ Florence, Italy.; ^6^ Centre for Research and Implementation of Clinical Practice, London, United Kingdom.

**Keywords:** lymphedema, lymphoedema, self-management, self-efficacy, children, adolescents

## Abstract

***Background:*** The aims of this study were to explore, using visual art methodology, how children and adolescents perceive their lymphedema and conceptualize the barriers and enablers in self-management and to explore the role of an educational camp in promoting self-efficacy.

***Methods:*** Participants (speaking English, French, and Italian) were recruited during an educational camp for children with lymphedema. Children and adolescents used different methodologies to depict living and self-managing their condition. Younger children (aged 5–12 years) drew pictures, and all children and adolescents (aged 5–18 years) were given cameras and asked to take photographs that depicted their experience of learning self-management of their condition during the camp. Rose's critical visual methodology framework was used for analysis.

***Results:*** Analysis of the data produced five categories: Normal versus altered childhood, living with lymphedema; perceptions of lymphedema and self-care in younger children; adolescents' perception of living and managing lymphedema; learning self-efficacy; and insights into cultural differences in self-care.

***Conclusions:*** The study has shown that self-management is complex. Children and adolescents face many daily challenges and frustrations in managing their condition in addition to the normal challenges of development and growth that impact on: home life, time with friends, school activities, and relationships. Children expressed a deep longing for cure and a recognition that their lives were altered by having the condition that led to limitations in sport and wearing fashionable clothes and shoes. The importance of relationships with professionals was critical as was the experience of meeting and learning with other children through the camp experience. Attempts to simplify self-management techniques would appear to be a key priority as would a greater understanding of the self-beliefs young people have of their ability to influence and control their condition and its impact on their life.

## Background

Lymphoedema is considered a rare disease in children and adolescents. The epidemiology of lymphedema in children and adolescents is poorly understood with current views indicating that 1 to 1.5 per 10,000 are affected.^1^ Lymphedema results from a complex array of developmental defects in the lymphatic circulation that occurs *in utero* with some occurring due to genetic abnormalities. More rarely, children may suffer with secondary lymphedema due to lymphatic damage from treatments for cancer or trauma.^2^ The clinical presentation may occur at any time up to young adulthood. As the limb increases in size, the term elephantiasis is used to define the skin changes such as papilloma and fibrosis that occur. These changes lead to functional impairment and are frequently accompanied by symptoms such as pain and paresthesia. Currently, the condition cannot be cured, although control is possible with effective treatment. Little is known of the impact on issues such as body image and normal development in these children.

### Treatment approaches

Treatment is based on a course of “intensive treatment” usually provided daily for several weeks involving skin care, exercise, manual lymphatic massage, and compression therapy. This is designed to reduce the residual excess fluid in the tissues and improve the skin condition and function of the limb.^3^ Following this phase, lifelong daily maintenance treatment using a modified version of these techniques is taught to parents and children to help prevent deterioration.

### Self-management in lymphedema

There is a growing emphasis in the health literature on the need for patients to effectively self-manage their long-term conditions.^4^ Although research into the experience of children and young people with lymphedema has shown the disease impacts on a range of life issues, including family life and goals,^5^ very little is known about how children and young people self-manage their condition. Indeed, there is very little consensus at present as to what constitutes self-management in lymphedema. There are large international variations in attitudes and practices held by lymphedema professionals involved in teaching families how to care for their condition.

It is now recognized that the lack of awareness and limited knowledge of lymphedema leads to delays in diagnosis and absent or inappropriate treatment regimens.^5^ Recent systematic reviews found few studies focusing on psychosocial issues and self-care regimens.^6^ The focus of much of the literature refers to aiding patients to “self-manage” without being conceptually clear on what this means.^7^ The literature frequently refers to compression, exercise, skin care, and massage rather than the wider issues and the importance of self-efficacy in promoting treatment adherence. Most articles on children with lymphedema focus on the clinical challenges of the disease.

The systematic review by Phillips and Gordon excluded studies, which looked at psychological support and treatment of children in adult services.^8^ In the eight studies they reviewed, they concluded that the research was generally of poor quality. The studies they looked at suggested that exercise is an important factor in the management of the condition but concluded that it is difficult to suggest how much is required. The review highlighted the challenges of outcome measurement in children, and the inappropriateness of using adult measurement techniques for children. They highlighted the complex array of treatments and the need to consider the overwhelming challenge for parents and children in understanding what it means to successfully “self-manage.”

What is striking from much of the existing evidence into self-management of lymphedema for children, adolescents, and parents is that much of the most current evidence says little more than Smeltzer et al. did in their 1985 review.^1^ It described examples of what other areas do, but not what should be performed. The focus remains within adult services, which describe the need for a multidisciplinary approach^9^ to teach self-management skills through individual and group education, but research remains sparse in terms of treatment programs that have an integrated approach. Authors still use the word compliance, which only serves to perpetuate ideas of parents and children who are not “doing what they know they should,” and is considered an inappropriate term when discussing self-management. They concluded that teams should approach lymphedema care in terms of a mutually agreed approach, which seeks strategies for patients that takes account of their lifestyle and family. The issue here is that there is little existing evidence of literature, which explores self-management treatment for children, adolescents, and young people with lymphedema.

### Self-management in children/young people with chronic diseases

Given the lack of evidence for lymphedema, it may be useful to consider self-management for children, adolescents, and young people with other types of chronic conditions. Jedeloo et al. conducted a Q-methodological study of adolescents with chronic diseases (semi-structured interviews) to discover their self-management and hospital care preferences.^10^ Their results demonstrated that there was no consistency in terms of what their preferences were. These ranged from autonomy and a “good life” to adherence to prevent future complications. They found that adolescents did not always want to know everything about their condition or prognosis but were more concerned with the implications for everyday life. Interestingly, adolescents were keen to have their parents support and involvement and referred to studies, which confirmed that adolescents found parents' encouragement important in terms of building their confidence to self-care. While the adolescents differed in terms of their desire to be involved in decision making, a key finding was the importance of being able to have their views heard and represented. The expectation of emotional support and a trusting relationship with health professionals was also referred to. They described four typologies of adolescents (“conscious and compliant,” “backseat patient,” “self-confident and autonomous,” and “worried and insecure,” p.598–599), and it is these which could be used as the foundations on which to develop programs or education to support children and young people. The study included adolescents with a wide range of conditions, which may be usefully applied to those suffering with lymphedema.

### Self-management and adherence to treatment

A systematic review of 42 self-management interventional randomized control trials, which focused on young people with chronic conditions, found that self-management interventions may influence an increase in knowledge and adherence to treatment regardless of the underlying disease or its severity.^11^ They concluded that individualized self-management interventions, both at home and within the clinical setting, may support adherence related to medical management, whereas a peer-support approach may be effective in terms of living with a chronic disease/condition day to day. This study raises the point that in implementing such interventions it is imperative that their effectiveness is evaluated. They advocated that a holistic approach is necessary, which combines online-peer support with an individualized self-management plan.

This review of the literature indicates the need for further studies, which explore the experience and range of care and treatment that children with lymphedema receive, combined with strategies that address the specific needs of children, adolescents, and young people and their families in terms of successful self-management of their condition. The research presented in this article forms part of a larger study using mixed methods to explore how self-management is conceptualized by different stakeholders.

### Aims of the study

The study has two related aims:

- To explore, using visual art methodology, how children and adolescents perceive their lymphedema and conceptualize the barriers and enablers in self-management.
- To explore the role of an educational camp in promoting self-efficacy in daily self-management regimens.

### Research setting

The children and adolescents recruited were attending an international educational camp for children with lymphedema in Turin, Italy, which was run under the auspices of an international charity the International Lymphoedema Framework (ILF), which is dedicated to improving care for this condition worldwide.

Children and adolescents were invited to attend the camp by lymphedema specialists from centers in France, Southern Ireland, Canada, Italy, and South Africa. No formal criteria for attending the camp were required, and children were selected for different reasons by the professional teams. Each family was able to bring their siblings, and the families were fully funded to attend. Language was not limited, and simultaneous translation allowed the team to undertake the research in the parents and children's native language.

### Patient sample and ethical issues

Children aged between 5 and 20 years were invited to attend the camp and thus to participate in the research. No clinical inclusion or exclusion criteria were applied other than a confirmed diagnosis of lymphedema.

The study was undertaken following the international research standards including the Declaration of Helsinki.^12^ Ethical approval in Italy was not required as this study was not an intervention study; however, ethical approval was obtained in the United Kingdom through the University of Nottingham Faculty of Medicine and Health Science Ethics Committee. All study information were translated and back translated into the different languages to ensure accuracy. French- and Italian-speaking parents reviewed the documents that had been translated to ensure that they were easily understood by the parents and children. We were mindful of the issues of gaining and maintaining consent with children and ensured that they were fully informed throughout the camp gaining verbal re-consent at each stage and ensuring that they knew they had the right to withdraw at any time for any reason.

All study documentation was archived, and pictures and photographs were anonymized and scanned into a secure encrypted computer only accessible to the research team. All photo data obscured the facial features to prevent identification of the child in publications. The research team decided to not identify within the reported visual data the child's country of origin to further protect anonymity.

## Methods

**FIG. 1.**
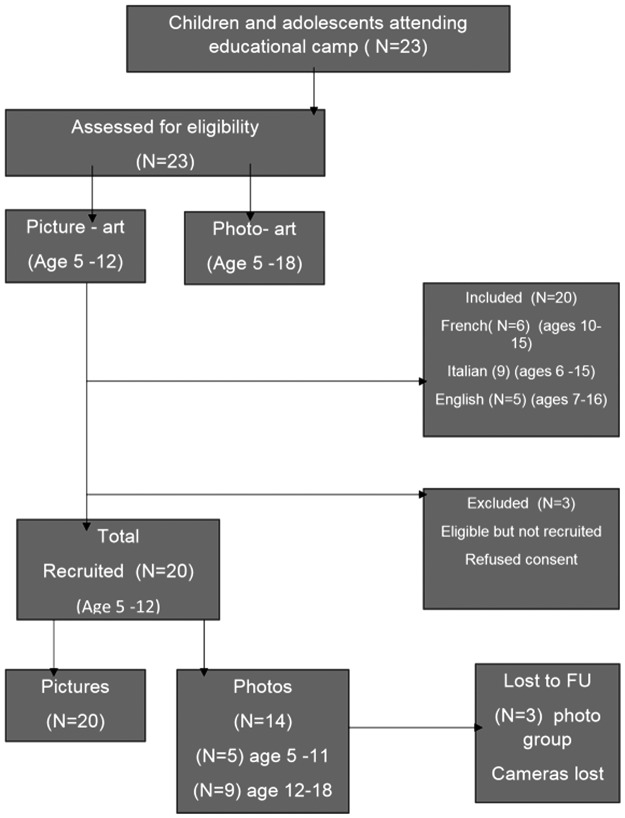
Flow diagram of children and adolescent participation.

Children and adolescents used different methodologies to depict their description of living and self-managing their condition. This is diagrammatically presented in Figure 1.

### Methods for picture art

The younger children (aged 5–12 years) were asked to draw a picture of living and managing their lymphedema. They were provided with paper, colored pens, and a range of stickers with different facial emoticons. While they were drawing their pictures, the research team discussed their drawings with them. The pictures were drawn on the first morning of the educational camp before they began to participate in the joint educational workshops with other children. Their siblings were also invited to join the exercise.

The children attending spoke three languages: English, French, and Italian. Information and instructions were provided to the families before the camp, and information sheets were provided again by researchers fluent in each language 24 hours before the research was undertaken. The same researchers assisted in helping to understand the images being drawn during the session, which lasted 90 minutes. Parents provided informed consent for pictures and images to be used in the research and future publications. Children gave additional assent to this process and were also given the option to take away their picture if they wished. The researchers asked children who wished to take away their pictures for consent to photograph them so that they could be included within the analysis.

The methodology used is based on the work of Guillemin,^13^ and analysis was undertaken using Rose's critical visual methodology framework.^14^ The methods were selected because of the difficulties of undertaking in-depth interviews with young children and the ability to encourage normal play activities to explore complex issues in a safe and fun environment, which would encourage engagement with younger children. The methodology provides a consistent approach in which to analyze the visual data.

Two independent researchers initially reviewed the pictures and asked the following questions about each picture:

- What information did the child provide about the contents of the picture and the reasons why the drawing was undertaken?
- What is being shown?
- What are the components of the image?
- How are the components arranged in the picture?
- What relationship, if any, is established between the components of the picture?
- What do the different components of the image signify and what do they represent?
- Is this a contradictory image (applied if several pictures were drawn and compared with images from the photo art)?

A further analysis was undertaken of the findings by a third researcher, and any issues of debate were discussed by the research team to aid clarification. Following the individual picture analysis, comparison was made across the whole data set to identify similarities and differences.

### Methods for photo art

Following the same informed consent procedure as described above, younger children and adolescents were provided with a digital camera at the opening session of the camp. They were able to keep the camera following the research. They were asked to take photographs of images that depicted their experience of learning to self-manage their condition during the camp. After the final session, photos were downloaded on to a secure database with a patient-protected encryption for each case. Families were asked to check all photos and remove any they did not wish to be included in the research when the photographs were loaded.

Duplicate images or obscured photos were withdrawn. The photos from each child were examined as a complete data set, which allowed a picture board for analysis to be collated. The Rose methodology was applied in the same was as for the picture art with three independent researchers.

### Comparison of picture and photo art

Following this stage of analysis, comparison of the pictures and photos were compared in several ways. Data from younger children who completed a picture and photographic picture board were analyzed as individual cases. Photo-data picture boards from adolescents were also analyzed as individual cases. Following this, data were compared across the subgroups (children, adolescents, and young adults) to identify the categories and finally compared across the whole data set to identify potential cultural and country differences.

## Results

During the camp, 23 children attended and were available for inclusion in the research.

Table 1 depicts their characteristics. Three children chose not to participate in the research and two children lost their cameras and were unable to upload their photographs.

**Table 1.** Characteristics of Children with Lymphedema

*Total group (*N = *20)*	*French speaking*	*Italian speaking*	*English speaking*
Number of children	6	9	5
Age range, years	10–15	6–15	7–16
Gender			
Female	4	7	2
Male	2	2	3

In total, 20 pictures were drawn and submitted by the younger children and 14 sets of photographs with 5 of these from the younger age group and 9 from the adolescents.

### Analysis of the combined data revealed the following categories

#### Categories

1. Normal versus altered childhood—living with lymphedema
2. Perceptions of lymphedema and self-care in younger children
3. Adolescents' perception of living and managing lymphedema
4. Learning self-efficacy
5. Insights into cultural differences in self-care

Each category will be discussed and supported through the presentation of the images and textual description obtained.

##### Category 1: normal versus altered childhood—living with lymphedema

**FIG. 2.**
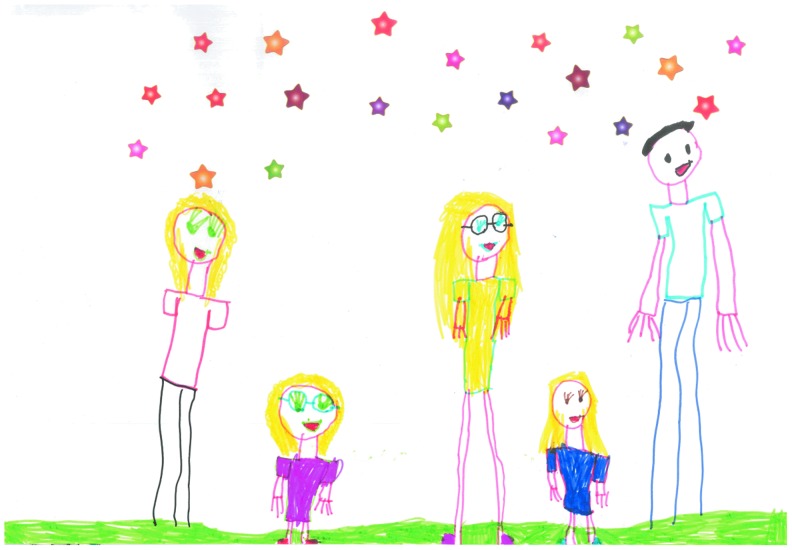
My family “this is my family all together we are happy.”

**FIG. 3.**
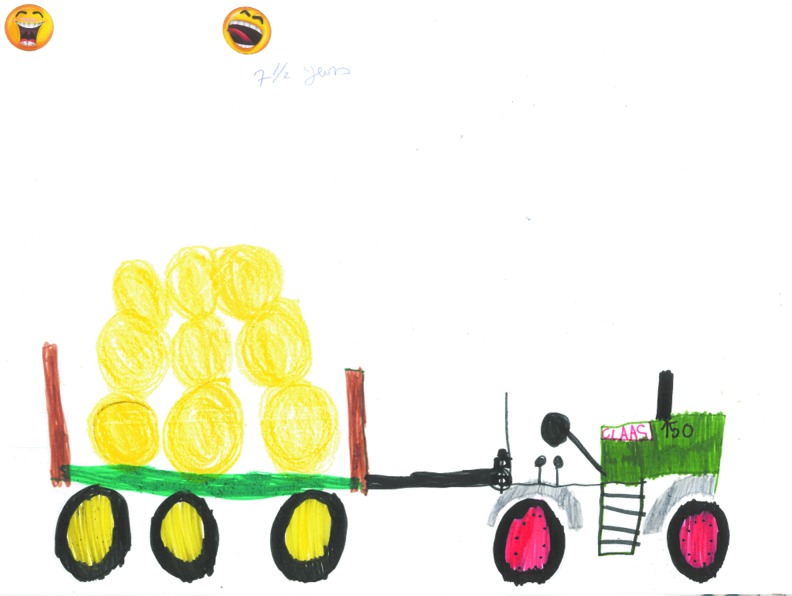
My favorite activity “this is what I like to draw being on a farm and seeing the animals and big tractors.”

The pictures drawn by the younger children frequently depicted normal childhood activities occurring with their families with a number choosing to draw pictures of their superheroes, activities they enjoyed, and being part of an adventurous holiday experience. These images made no reference to lymphedema or its impact on the child or family. A typical example of these type of images is depicted in Figure 2 drawn by an 11-year-old girl who described the image of her family and Figure 3, a boy of 7 who drew his favorite activities.

**FIG. 4.**
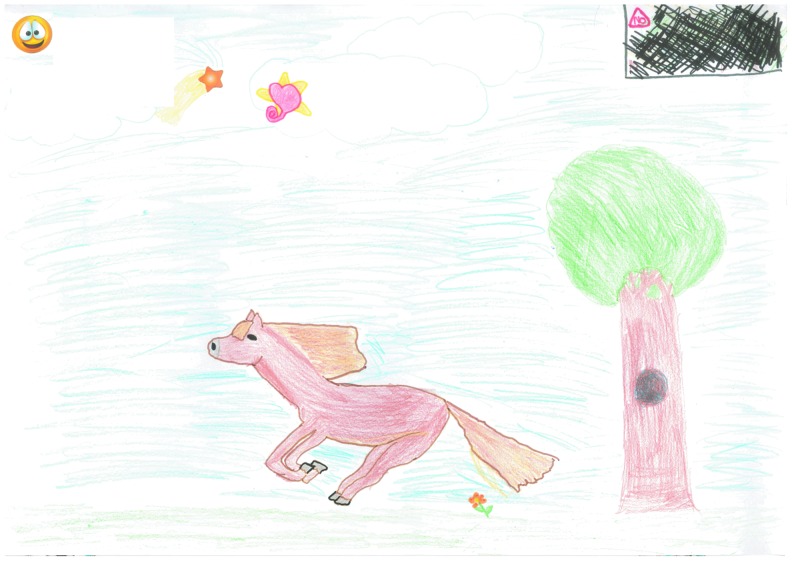
The impact of lymphedema on life “I like to draw horses but I don't like my Lymphoedema so I drew it in the corner of my picture because it makes me cross sometimes.”

Four children referred to the influence of lymphedema on their daily life. Figure 4 was drawn by a 12-year-old boy who chose to represent the impact of lymphedema on life by drawing a black box in the corner of the picture. The use of strong color and images suggested not only the presence of lymphedema but also an insidious and difficult part of this child's life.

**FIG. 5.**
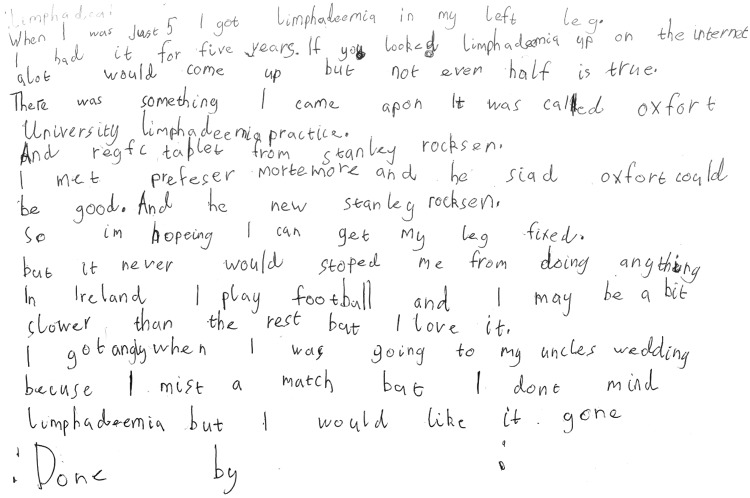
Writing about my lymphedema “I don't want to draw you a picture I will write about my Lymphoedema for you because I like writing.”

A 10-year-old male chose to write a dialogue about his lymphedema that reflected his knowledge of his parent's anxiety about obtaining a correct diagnosis and treatment and a search for a cure. The same child expressed the desire to push the boundaries of childhood and embrace more risky activities as was demonstrated in his photo art. A short extract of his writing is depicted in Figure 5.

Some children were accompanied by siblings who also were able to draw pictures. These did not include any reference to lymphedema.

**FIG. 6.**
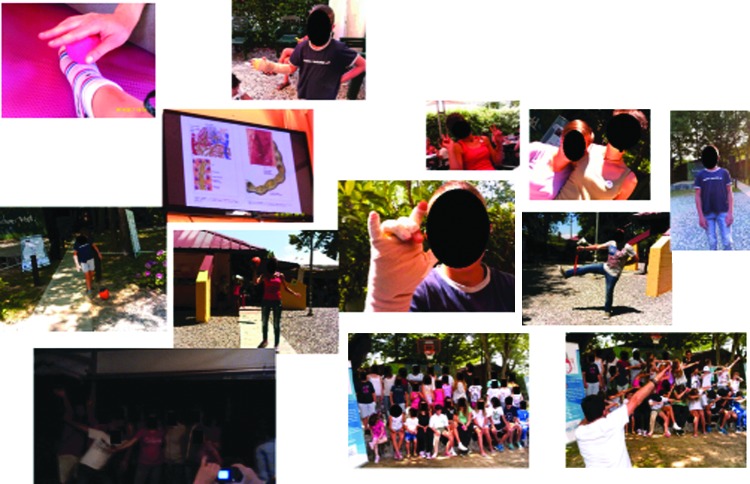
Living and managing my lymphedema.

**FIG. 7.**
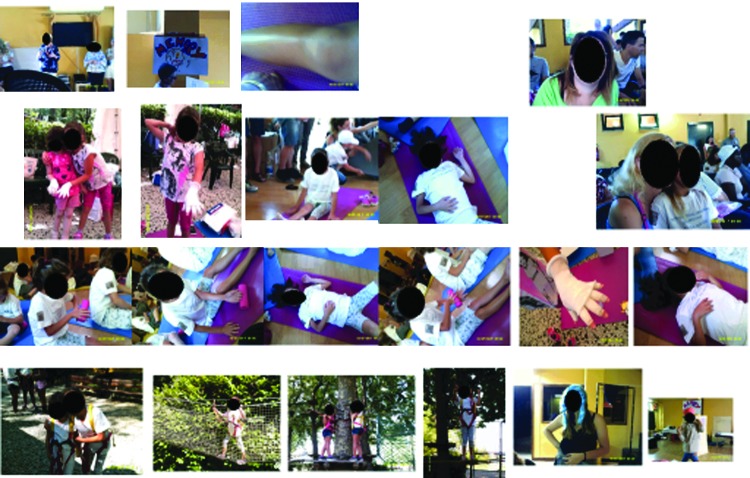
Learning about my lymphedema with my friends.

**FIG. 8.**
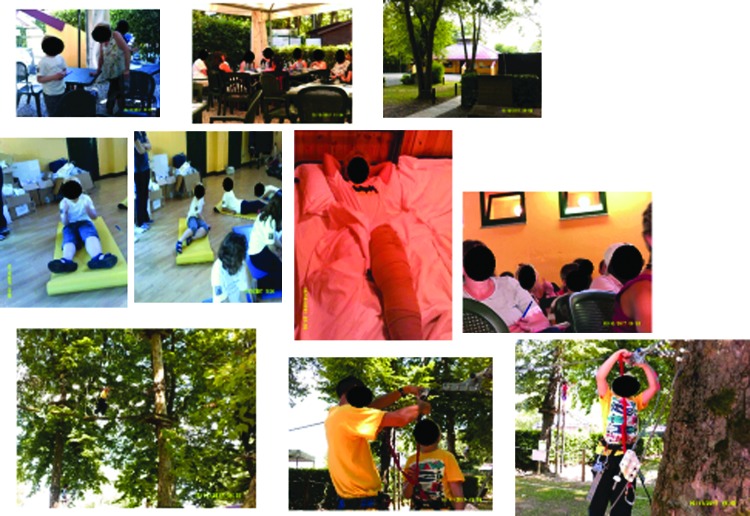
Learning to cope with my lymphedema together at camp.

Analysis of the photo art also supported the importance of normal childhood activities that were enjoyed with the family and the development of new relationships formed during the camp experience and of undertaking challenging sports activities. All the photo collages demonstrated the importance of the camp in shared experience during the educational workshops (Figs. 6–8). The importance of access to a professional clinical team and the relationship of trust with them were evident for younger children and adolescents. All but one child recorded self-management in group activities. The child who chose not to display this may have been influenced by other more profound health issues, of which lymphedema was not a priority. This child photographed food and family activities and drew a picture of a heart.

Figures 6–8 shows the photo collages of camp activities.

**FIG. 9.**
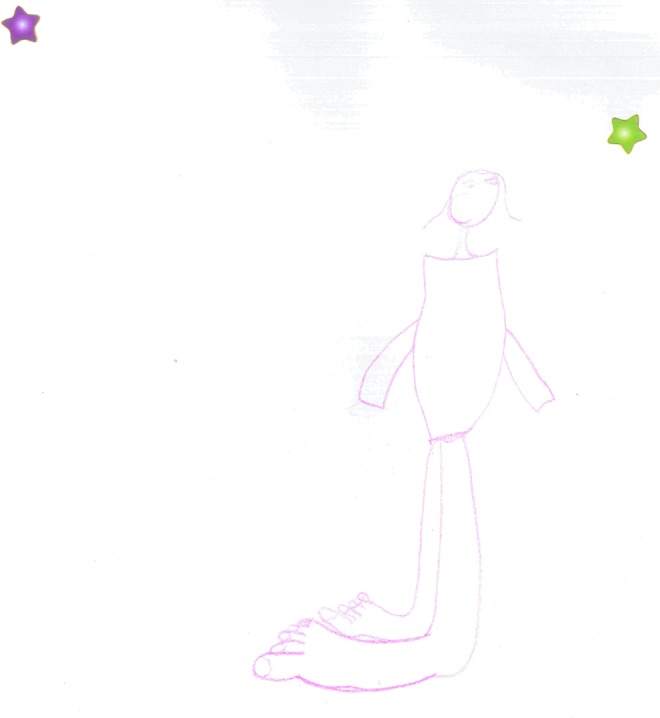
Depiction of leg lymphedema “this is a drawing of my Lymphoedema.”

**FIG. 10.**
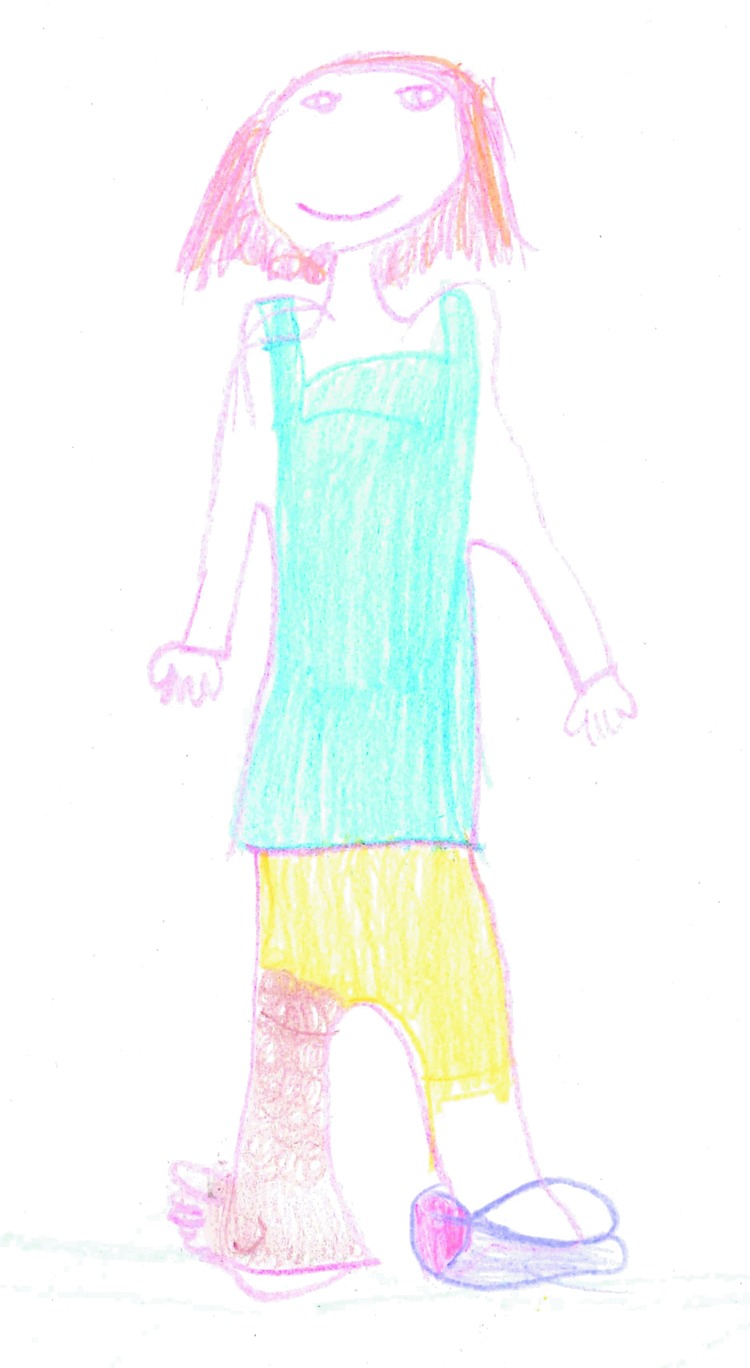
Wearing a compression bandage “I have to wear a bandage every day and here is a picture of me wearing it. Sometimes it makes me sad but today I am happy.”

Three children chose to draw images of themselves and their condition. Figure 9 shows the exaggerated proportions the child has of their lower limb lymphedema, and Figure 10 depicts the wearing of a bandage as part of treatment.

A striking difference emerged between the pictures and photo art. The pictures drawn by the children who chose to draw their lymphedema depicted them alone rather than within a family unit suggesting the isolating experience of living with lymphedema. In contrast, the photo art showed the importance of collective experience with both the family and other children affected with the condition. Many of the children had not met a child with a similar condition before attending the camp.

##### Category 2: perceptions of lymphedema and self-care in younger children

**FIG. 11.**
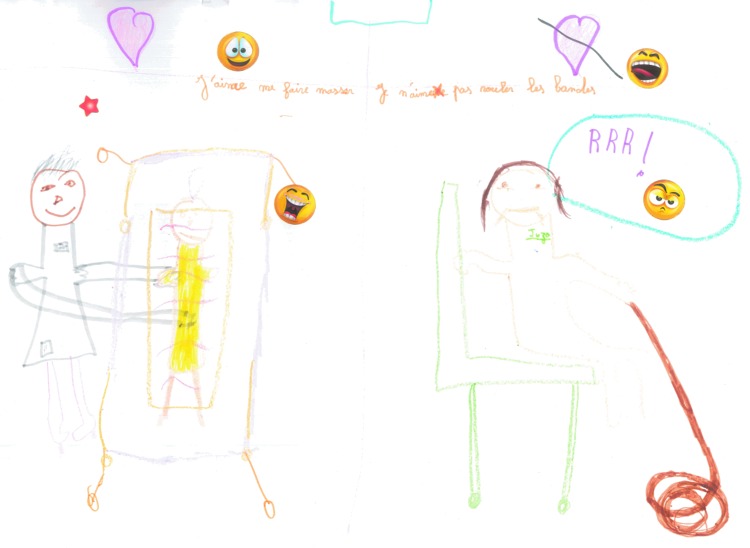
*Left image*—enjoying the relationship with the therapist. *Right image*—frustration of managing compression bandaging. “I like (X therapist) she is kind and sometimes she tickles me and makes me laugh. I don't like my bandages and having to roll them up. Sometimes I am sad and cross.”

**FIG. 12.**
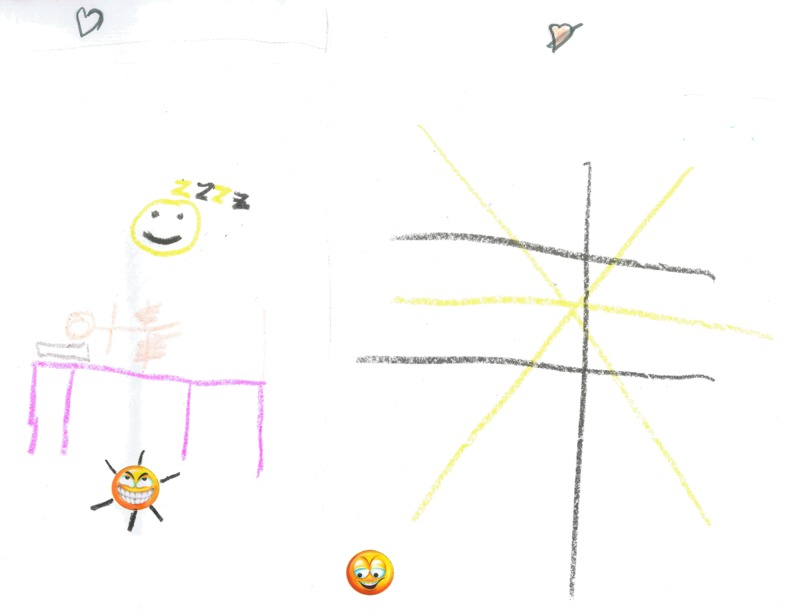
Daily treatment is intrusive and frustrating. “I like to go to sleep but my Lymphoedema makes me sad because I want to play.”

Children were able to use pictures and photo art to depict their perceptions of self-care. Three younger children drew pictures of having treatment that expressed the importance they placed on the relationship they had with their therapist but additionally the daily intrusion that self-management placed on their lives. Figures 11 and 12 show strong images with the use of color and negative emoticons to describe their experiences. Two children referred to the fact that treatment impacted on sleep suggesting the intrusion of self-treatment. Figure 12 shows the extreme frustration experienced.

The photo art captured the experience of learning new self-management techniques together with other children and experimenting with new materials.

##### Category 3: adolescents' perception of living and managing lymphedema

**FIG. 14.**
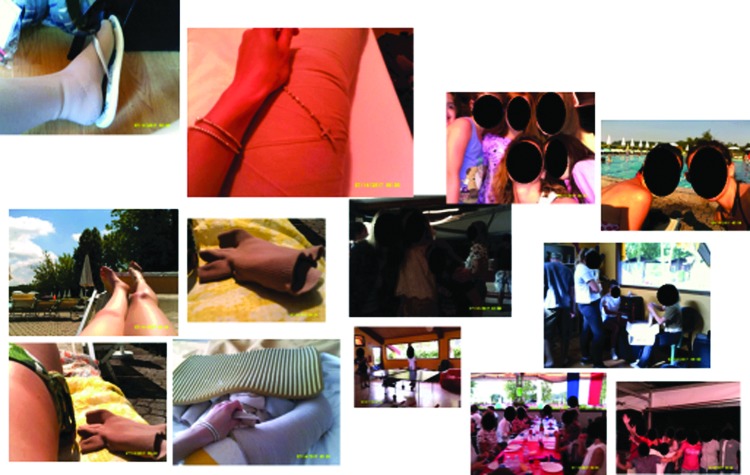
The difficulties and intrusion of self-care regimens for adolescents. “I can never get away from my Lymphoedema I have to think about it all the time every day and work really hard to stop my leg from getting bigger which it was at the beginning. It's like you can't escape.”

The adolescents also were able to depict their experience of managing their lymphedema and the implications this had on daily life. The photo art showed strongly the importance of belonging to a peer group. The additional interview with a young female revealed the search for a normal limb shape so that she could fit in with her peer group. A spectrum of reactions and attitudes to self-management were found. Some were ambivalent to the parental wish for them to undertake daily routines, while one young adult showed extreme vigilance to self-care to prevent deterioration and a return to how her leg had been before treatment. Figure 14 shows the fact that self-management is pervasive and involves all aspects of daily life. Even normal activities such as sunbathing require modification with limb elevation, and the compression hosiery is depicted within the same image. Adolescents were prepared to take pictures of their affected limb and of wearing compression bandaging. In a similar way to the younger children, there was evidence of the importance of shared learning together and the importance of being a role model and mentor to younger children. Adolescents were able to depict their hope for the future and how they used coping mechanisms such as turning to religion in their quest to manage and contain their condition. It should be noted that three teenagers chose not to engage with the use of photos indicating that this is a complex issue that adolescents may find too difficult to easily describe within such a program.

##### Category 4: learning self-efficacy

**FIG. 15.**
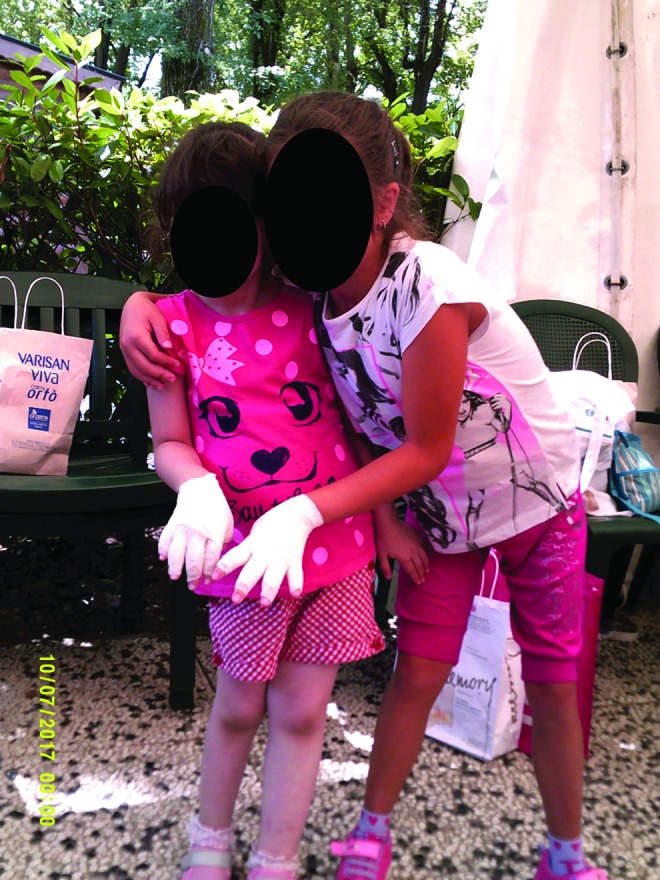
Younger children learning to bandage together.

**FIG. 16.**
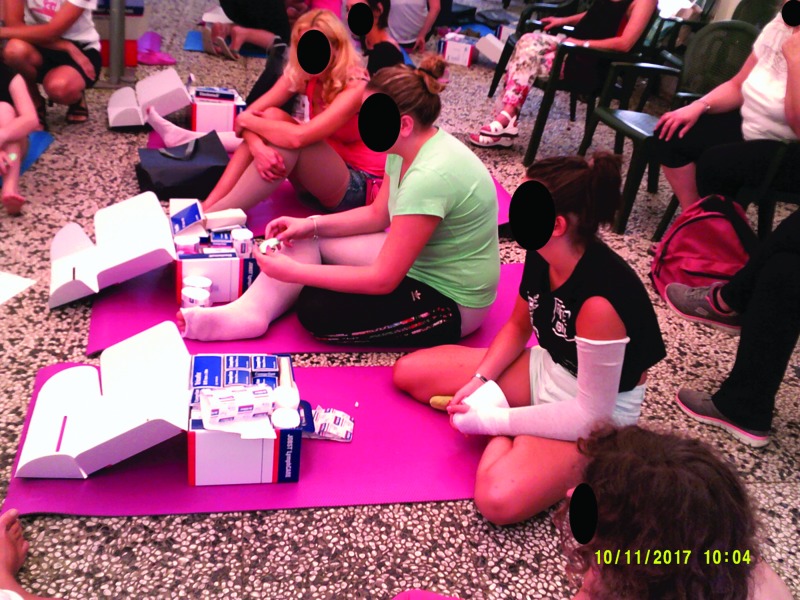
Adolescents practicing the application of compression therapy.

**FIG. 17.**
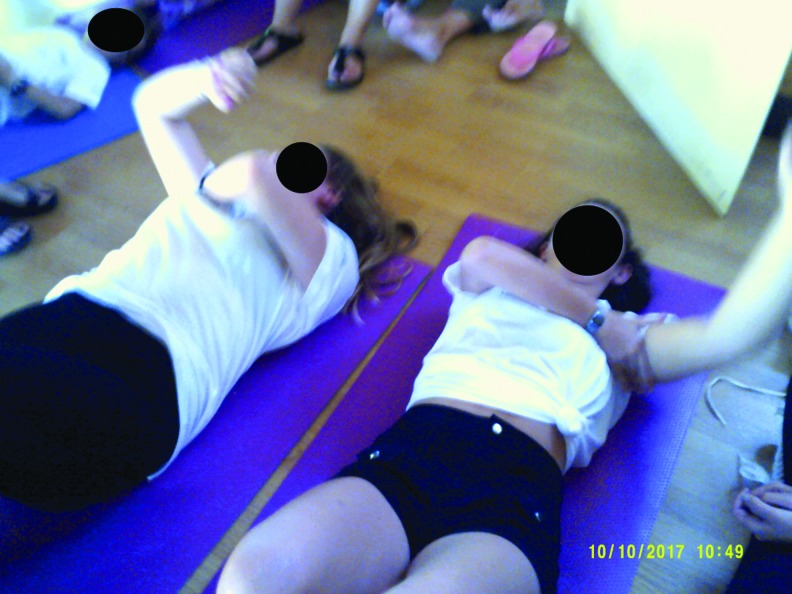
Practicing manual lymphatic drainage techniques together in a workshop.

**FIG. 18.**
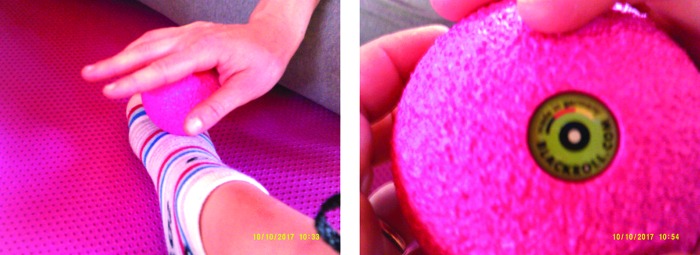
Using newly developed devices in treatment.

The photo art suggested the camp experience was enabling the development of improved self-efficacy in children and adolescents who were participating in the educational workshops. The photos indicate their emotional engagement with learning and suggest that this represents a new “norm” was established for the group. Younger children (Fig. 15) chose to record how they bandaged each other and collectively learned the techniques. Adolescents also chose to photo these joint activities, Figures 16 and 17 depict examples of this. New novel devices for undertaking massage were also photographed in use (Fig. 18).

**FIG. 19.**
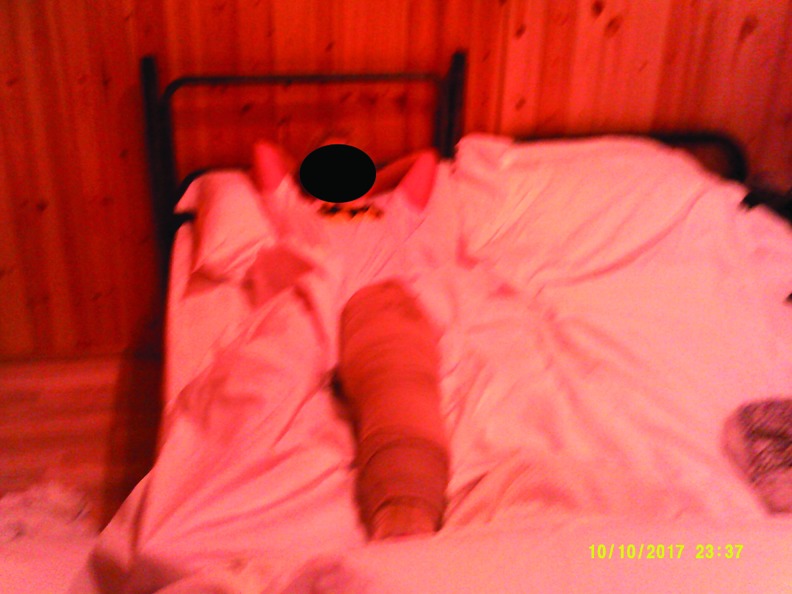
Child who had refused to wear compression bandaging before the camp.

In addition to more formal activities, fun activities of swimming and climbing were prominently displayed and group activities such as football and other games. One child who refused to wear compression before the camp started to undertake this himself and records this in an image of himself (Fig. 19).

The photo art demonstrated the importance that the groups felt of having access to skilled professionals to assist them in their quest. Pictures of the therapists teaching and sharing with them were frequently used in addition to pictures of the research teams working with the children and families suggesting that the research process may itself form an intervention that may help children and families understand their condition and to provide a voice to their experience that may not naturally be available within clinical services.

##### Category 5: insights into cultural differences in self-care

**FIG. 20.**
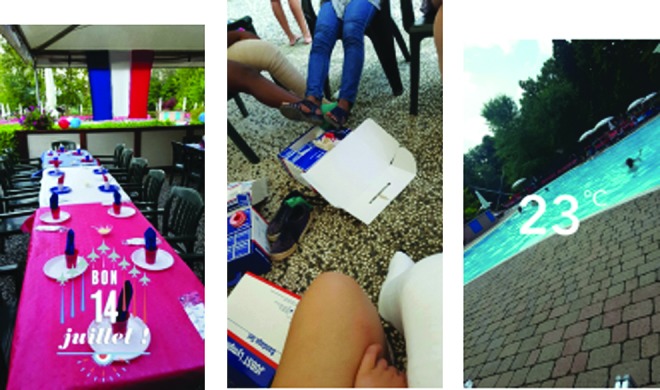
Evidence of the importance of belonging to a national group.

There was evidence of the importance and pride of being connected to a larger group of parents and professionals within the services that were represented. The French team had a particularly strong cohesion that was frequently celebrated during the social events as demonstrated in Figure 20. This was also influenced by the celebration of Independence Day during the event. The Italian families and therapists were also very collective in their relationships despite them coming from different therapy-led services and the lack of a specialist multidisciplinary service. Parents and children who spoke English quickly established relationships with the other parents and children despite the difficulties of language. Very quickly, the children coalesced within the joint activities together.

## Discussion

This study set out to explore, using visual art methodology, how children and adolescents perceive their lymphedema and conceptualize the barriers and enablers in self-management and the role of an educational camp in promoting self-efficacy. Five categories emerged from the combined data: Normal versus altered childhood—living with lymphedema; perceptions of lymphedema and self-care in younger children; adolescents' perception of living and managing lymphedema; leaning self-efficacy; and insights into cultural differences in self-care.

The impact of living with lymphedema presented a complex picture. Younger children rarely represented their lymphedema in their art, but when they did, there was evidence it had a subtle but pervasive effect. Research on coping with chronic illness often refers to the stigmatizing effect of a condition and the way this influences people's ability to adapt and integrate into normal life. The search for a “normal” life was prevalent in this research. Ferguson and Walker in a qualitative study of adolescents with chronic illness found that living normally was important and required managing a level of risk and developing resilience in the face of challenges.^15^ Children and adolescents in this study spoke of their hope for a future cure and were aware that this was important to their parents. Control of the swelling was not easily achieved and required persistence with treatment and a belief in their own ability. This was often at a significant physical and emotional cost. Research with adolescents with chronic illness has defined the “chronic sorrow” and “living loss” as part of this journey that requires adaptive coping skills to prevent psychological distress and the relief of isolation.^16^ The journey into adulthood required adolescents with lymphedema retained hope that they could manage and control their condition; hope has been recognized to be important for adolescents suffering with other chronic illnesses.^17^ It is an essential human strength that has been shown to promote health, facilitate coping, improve quality of life, and increase self-esteem and personal resilience.^18^

**FIG. 13.**
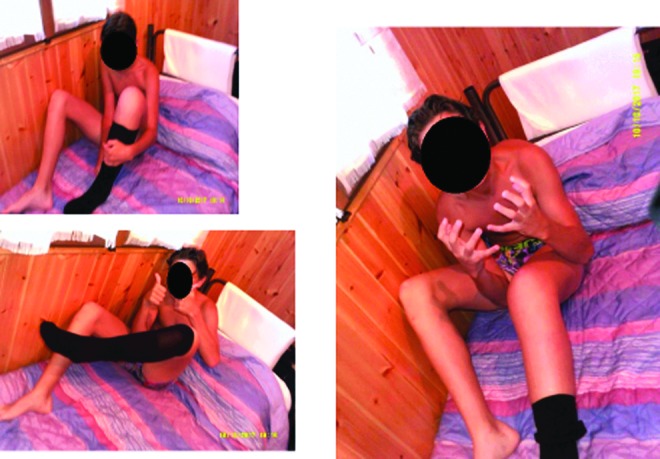
The frustration of managing my lymphedema.

Many of the children in this research had never met another child with the same condition. The sharing of the workshops created a new “norm” for them to learn together and helped relieve the isolation they felt. Research during a therapeutic camp for children with chronic illness showed an improvement in quality-of-life status when measured before and after the event. This occurred irrespective of age, gender, and the type or severity of the disease.

This study explored the issues of self-management with lymphedema treatments that require a daily adherence to practical techniques, such as bandaging and exercise. Some of the children and adolescents were seeking greater self-efficacy in addressing this. However, this was not a uniform finding with evidence that some were resistant to engagement with treatment despite their parental support and encouragement. This suggests that they were all at different stages of using self-efficacy beliefs ranging from nonadherent behavior to motivated long-term adherence.^19^

Social cognitive theories have been developed to help explain how self-efficacy is linked to health behavior. The Theory of Planned Change indicates that intention is the most important predictor of a change in health behavior through a range of cognitive processes including the following: the attitude to the behavior; the subjective norm (the views about the behavior held by those close to them); and the perception of control.^20^ Bandura's research also supported that when people have a sense of control they can control their behavior and that self-efficacy should be considered synonymously with behavioral control.^21^ The Transtheoretical model defines a five-stage process beginning with a stage of precontemplation to a final stage of acceptance when the behavior is embedded in daily life and also suggests a further step, which is reached when there is no residual temptation to give up.^22^ Other research such as the Health Action Process Approach focus on the mechanisms that occur after the intention to change has been made.^23^ These approaches indicate that this is a complex issue that has not been addressed in children or adults facing the challenges of managing lymphedema.

Lorig and Holman explored the origins and limitations of self-management research and measurement.^24^ They referred to the limitations of approaches that are limited to a medical model, which fails to take account of the processes required to become effective in self-management. They recommend that approaches should include activating resources, helping them to learn to live with the condition, and developing the tasks and skills required. There is evidence in this research that lymphedema self-management is approached largely through a medical model that incorporates clinical techniques.

### Methodological challenges in use of visual art methodology

Guillemin^13^ in his review of visual research methods suggested that drawings are well suited for use with young children who are not able to fully articulate the emotions and beliefs they have about a subject. He argued that drawings in a similar way to other forms of visual imagery are about how people see the simplicities and complexities of the world and help to elicit meaning. However, production of visual imagery is influenced by many issues, such as power relations and social experiences. Production of visual images is not considered to be fixed or static entities. Drawings and photographs produced in this research were produced at a fixed point in time and may not be reproduced in the same way at another time. The production of the visual images will have been influenced by the children's past, their present experience, and their future aspirations. The images therefore, like other representations, can be used to understand how the children and adolescents saw their world and experience of lymphedema at fixed time and place.

Rose in the methodological description of the approach taken in this research stresses a number of important issues.^14^ The visual images required the participants where possible to reflect and discuss the image they have drawn or photographed. This included questions about how the drawings and photos made them feel and why they chose colors or symbols such as emoticons. This may be challenging for younger children to undertake due to their lack of cognitive development to understand the reasons behind the choices they have made.

In addition to the assessment of the image produced, it was important to consider other aspects:

- The context of the images and when they were produced during the camp program.
- The level or familiarity and relationship between the children and the research team who had not previously met the children or young people before the camp.
- The ability and willingness of the child to produce the image and whether assistance from siblings or other children was used.
- The relationship of the person creating the images.
- The clarity in which the children understood the task they were asked to perform and what types of clarification or help were required during the session to enable the children.
- An understanding of any technical issues that may arise from the children and adolescents in the use of the digital cameras.

Guillemin^13^ when discussing the use of visual imagery of women's heart disease noted some were unable to either draw or write their experience and that this was a reflection that meaning about illness is often based on the use of words rather than the use of images. The women used metaphors and symbolic representations such as broken or damaged hearts in a similar way to the choices made by the younger children in this study who also used symbols of hearts and different facial emoticons to represent their feelings. The drawings in this study often used dark colors to reflect negative emotions as was also seen in Guillemin's research.

Kirova and Emme^25^ explored the methodological challenges of using still photographs within a phenomenology-based study of children experiencing immigration. They defined the use of photography in this study as “a form of capturing and communicating the unspeakable in an experience (p.3).” They discussed the ability of a photograph to capture nonverbal communication that are impossible to achieve within verbal data while recognizing that all forms of data are incomplete and tell a partial story.

While there are potential limitations in the use of drawings and photographs and a continuing reliance on word-based research methods, nevertheless these methods offer a means of gaining a deeper insight into the ways in which children and adolescents represent the challenges of living with a chronic condition such as lymphedema and the daily issues of self-management.

### Limitations

There are limitations to this study. Not all adolescents participated, and it was not possible to determine the reason why they did not engage with the process. An educational camp brings together a large group of people who need to establish relationships quickly to engage in group activities. This may be particularly challenging for adolescents who do not come with an established group and may also be influenced by the natural barriers of language. It is well recognized that people who attend support groups are not representative of the wider population and therefore caution must be taken to apply these findings to all families with lymphedema. In addition, there is a selection bias as the criteria used by the professionals from the different countries in bringing children will differ. This is a cross-sectional study, and there would be significant value in a longitudinal study using these methods to explore whether changes in perception are occurring. The study also required the use of simultaneous translation services, which are expensive to employ and require careful interpretation of the data. It was envisaged that the process of translation may inhibit the flow of discussion, but this did not occur. The decision to engage the younger children in drawing pictures at the beginning of the camp helped to ensure that they were not significantly influenced by the experience of the camp activities. However, it may have led some children to be shy with researchers they had not had time to establish a relationship with.

It is also important to recognize that the children were attending a camp with their families and perceived this as a holiday experience. It is not possible to know whether the new approaches to care they learnt during the camp will be retained when they return to their day-to-day lives.

## Conclusions

The study has shown that self-management of lymphedema for children and adolescents is complex and that visual art methods can capture some of the dilemmas that cannot easily be described through other research methods. For many of the children, lymphedema was not prominently displayed within their artwork, particularly in the younger age group. However, there was evidence that there are many challenges they face in the daily routine of self-management that causes frustration and impinge on daily life for children and adolescents.

The importance of relationships held with professionals was critical for children and their parents and was able to offset some of the difficulties they faced. However, therapy impacts on family time and this is keenly felt by the children. Adolescents attending the camp were diverse in their willingness to engage with this type of study. Those who participated provided an insight into the relentless challenge to prevent the lymphedema worsening and the limb increasing in size. They also showed they viewed themselves as being useful advocates and mentors for younger children. While this study did not seek directly to understand the beliefs that children and adolescents had about their condition, there was evidence that many held a deep longing for the possibility of a cure and a recognition that their lives were altered by having the condition and this led to limitations in sport and wearing fashionable clothes and shoes.

The study has shown that self-management for children, adolescents, and parents is complex. Traditional individual approaches to learning should be challenged, and approaches that draw children, parents, and professionals together may be beneficial and inform the development of new therapeutic approaches.^26,27^

Children and adolescents face many frustrations in managing their condition in addition to the normal challenges of development and growth. Attempts to simplify self-management techniques would appear to be a key priority as would a greater understanding of the self-beliefs young people have of their ability to influence and control their condition and its impact on their life. While the emphasis in this work has been on the experience of the child and adolescent with lymphedema, there is a poignant silence in our understanding of the experience of siblings coping with this situation, which must also be addressed through further research.
